# Towards Improving TSCH Energy Efficiency: An Analytical Approach to a Practical Implementation

**DOI:** 10.3390/s20216047

**Published:** 2020-10-24

**Authors:** Marcos A. Sordi, Ohara K. Rayel, Guilherme L. Moritz, João L. Rebelatto

**Affiliations:** 1 PPGSE, Universidade Tecnológica Federal do Paraná (UTFPR), Curitiba, PR 80230-901, Brazil; marcos.sordi@ifpr.edu.br (M.A.S.); moritz@utfpr.edu.br (G.L.M.); 2 CPGEI, Universidade Tecnológica Federal do Paraná (UTFPR), Curitiba, PR 80230-901, Brazil; jlrebelatto@utfpr.edu.br

**Keywords:** TSCH, guard time, energy efficiency, synchronization

## Abstract

The IEEE 802.15.4-2015 standard defines a number of Medium Access Control (MAC) layer protocols for low power wireless communications, which are desirable for energy-constrained Internet of Things (IoT) devices. Originally defined in the IEEE 802.15.4e amendment, the Time Slotted Channel Hopping (TSCH) has recently been attracting attention from the research community due to its reduced contention (time scheduling) and robustness against fading (channel hopping). However, it requires a certain level of synchronization between the nodes, which can increase the energy consumption. In this work, we implement the Guard Beacon (GB) strategy, aiming at reducing the guard time usually implemented to compensate for imperfect synchronization. Moreover, besides presenting a realistic energy consumption model for a Contiki Operating System-based TSCH network, we show through analytical and practical results that, without the proposed scheme, the power consumption can be more than 13% higher.

## 1. Introduction

Energy efficiency is a mandatory requirement in the scope of Wireless Sensor Networks (WSNs), since these kind of networks are usually composed of battery-powered devices, whose batteries are not always easily replaceable [1,2]. For instance, one can mention either the lack of natural and economic resources or the harsh environments where many of such WSNs are placed as impairments for battery replacement [3]. Thus, it becomes of fundamental importance to spend efforts towards reducing the network energy consumption, extending its lifetime as much as possible [2].

The power consumption of massive Internet of Things (IoT) hardware is mainly attributed to the Microcontroller Unit (MCU) and to the transmitter/receiver chains. Many of the existing low-power radio transceivers typically operate with ~30 mW when in the listening mode, with a slight consumption increase when transmitting. However, regardless of the operating mode, the transceiver-related consumption turns out to be much higher than the MCU consumption [4]. Moreover, the software implementation is usually optimized to keep the MCU sleeping when it is not required. However, low-power radios require energy-concerned Medium Access Control (MAC) protocols. Traditional MAC protocols, such as Carrier Sense Multiple Access (CSMA), require uninterrupted channel sensing and are consequently not suitable for energy-constrained devices, being capable of completely draining the devices’ battery in a matter of days if not associated with Radio Duty Cycling (RDC) mechanisms [5].

The Time Slotted Channel Hopping (TSCH) scheme [6] reduces consumption by applying a time-scheduling approach that allows the nodes to awake only when necessary. However, it requires a certain level of synchronization between the nodes in order to avoid communication outages, since it is critical to guarantee that the packets arrive at the receiver when not in sleep mode. One alternative to mitigate imperfect synchronization-related issues is by adopting a guard time. Moreover, synchronization deteriorates over time due to clock drift, such that there is a need to periodically resynchronize the nodes [7]. Different approaches are adopted in synchronized MAC protocols to address the synchronization problem, such as increasing the Enhanced Beacon (EB) rate [8] or setting a larger guard time for the receiver [9]. Nevertheless, such approaches require either more message exchange or a higher RDC, thus increasing the energy consumption.

In [10] it was shown that the idle listening during the guard time is responsible for most of the overall energy consumption. In [11,12,13], the authors performed empirical optimizations on the guard time in order to maximize the energy efficiency in both single-hop and multi-hop TSCH Network, by adapting the guard time at each node according to its distance to the sink and a target packet delivery ratio. However, the authors rely on very small values for the EB Interval (TS=3.42s), which has the drawback of increasing the network traffic and being less energy efficient.

In [8,14], the authors propose a new beacon advertising approach for a fast synchronization. More specifically, although the scheme in [8] achieves faster joining times, the energy consumption is compromised in the initial deployment stage. Later, the same authors present in [14] a scheme that dynamically modifies the transmission period of the Enhanced Beacons, which can be properly adapted to reduce the power consumption and improve connection time and connection success rate. In [15], the existence of collisions between EBs is taken into account, and a novel autonomous EB scheduling method that eliminates collisions is proposed.

In [16] the authors propose an approach where the node proceeds with the re-synchronization by considering multiple routing parents from the IPv6 Routing Protocol for Low Power and Lossy Networks (RPL) as time source. The results suggest that, with two time sources, the network is able to maintain synchronization with smaller guard times, since the maximum synchronization error is contained. However, the authors consider very small values for the EB Interval (TS=10s), which, as previously discussed, is not efficient in terms of network traffic and energy consumption.

In [9], the authors proposed the so-called Guard Beacon (GB) method, aiming at reducing the guard time and consequently increase the energy efficiency of the synchronization process. In each synchronization round, a node sequentially sends several beacons, increasing the probability that at least one of them will be successfully received by the node to be synchronized. In a TSCH-based network, nodes send Enhanced Beacons in a periodic fashion [6], which are special TSCH packets that contain all the necessary information for a node to join the network and establish communication to the other nodes. Synchronism between nodes is then maintained by means of periodical EBs exchange to calibrate their clocks. Thus, employing the Guard Beacon strategy could lead to energy savings on a TSCH network, since the probability that at least one of the beacons is received is higher, which enables synchronism maintenance even with smaller guard times.

An Operating System (OS) designed for constrained IoT devices has some requirements in terms of Random Access Memory (RAM) and Read Only Memory (ROM) consumption, multitasking, strict power management and real time behavior [17]. Several operating systems have been developed to comply with WSNs requirements, such as TinyOS [18], RIOT [19], OpenWSN [20], Zephyr [21], Contiki [17] and many others. Recent works have evaluated the energy consumption of a TSCH-based network on different hardware platforms and operating systems [10,22,23]. An analytical energy consumption model for the OpenWSN TSCH implementation was derived in [23], being supported by experimental results. Based on [22], an improved model with a more up-to-date set of time slots and states was proposed, also adopting more recent hardware and firmware, and whose output accurately matches the experimental results.

Contiki is a very popular OS within the community, since it has a low memory footprint, implements several standards, has a good hardware support and is backed by both industry and academia, with more than 2000 forks, almost 500 watchers and 3000 stars on its official GitHub repository [24] (Codebase: https://github.com/contiki-os/contiki). Additionally, it has a TSCH and IPv6 over the TSCH mode of IEEE 802.15.4e (6TiSCH) implementation [5], a full low-power IP networking (IPv6 over Low-Power Wireless Personal Area Networks (6LoWPAN), RPL, Constrained Application Protocol (CoAP), and Message Queuing Telemetry Transport (MQTT)) and tools for software-based energy estimation (Energest and Powertrace [25]). However, by employing the Contiki OS together with its Powertrace [25] and Energest power profile, one can notice that the energy consumption of the nodes on a TSCH-based network is significantly different from the analytical models from [22,23]. Additionally, as reported in [10], the guard time length needs to be carefully adjusted aiming at properly satisfying the inherent trade-off between reliability and energy consumption. Finally, to the best of our knowledge none of the aforementioned works proposed a beaconing mechanism focused on guard time reduction. Thus, the contributions of this paper are twofold:

- We resort to the Guard Beacon strategy aiming at reducing the guard time of a TSCH-based network. Our results indicate that the standard Contiki’s TSCH implementation [24] power consumption is up to 13.05% higher than when our scheme is implemented;
- We perform a set of measurements on the Contiki’s TSCH timing, which allows us to provide a more updated set of time slots and states then the ones from [22], also including the Guard Beacon strategy. The model accuracy is verified by comparing the analytical results to the ones obtained from the Contiki’s Powertrace and Energest tools, which presents a close match regardless of the guard time length or the packet sizes.

The rest of this work is organized as follows. Section 2 presents the IEEE 802.15.4.e standard and its operating principles. The proposed energy consumption model is presented in Section 3. Section 4 presents and discusses some analytical and experimental results for a Texas Instruments CC2650 Launchpad hardware platform. Finally, Section 5 concludes the paper.

## 2. Time Slotted Channel Hopping—TSCH

**Figure 1:**
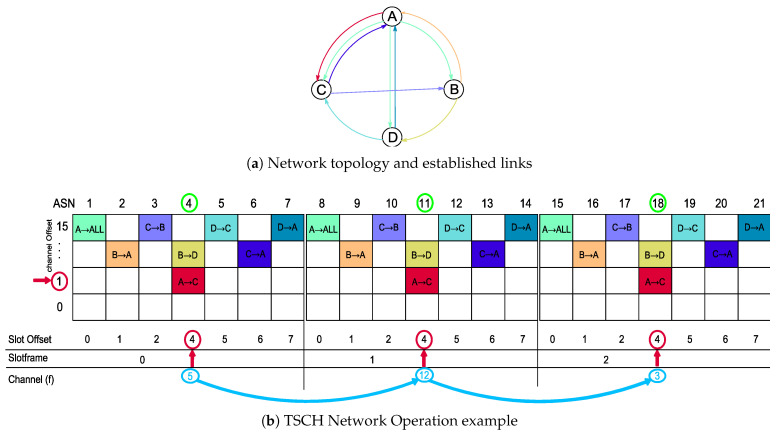
Network topology and established links (**a**) and TSCH Network Operation example (**b**). The 1st timeslot is dedicated to broadcast data: A → ALL, while on the following timeslots only unicast transmissions/receptions are performed. The slotframe contains 7 timeslots and 3 cycles are shown. The frequency is obtained from (1).

The Time Slotted Channel Hopping scheme is introduced in the IEEE 802.15.4e standard, aiming at increasing reliability and robustness [6]. In a TSCH-based network as the one illustrated in Figure 1, each transmitter–receiver pair is assigned with a fixed-size timeslot, in a Time-Division Multiple Access (TDMA) fashion that follows a predefined schedule. Moreover, there are $N_{c}=16$ independent channels available for communication, where the channel index *f* assigned to establish a connection is pseudo-randomly determined as

(1) $$f=F_{\mathrm{mapp}}\left[\left(\mathrm{ASN}+ch_{\mathrm{offset}}\right)\;\;\mathrm{mod}\;N_{c}\right],$$

where $ch_{\mathrm{offset}}$ is a parameter that allows different channels to be used at the same timeslot for different slotframes, ASN stands for the absolute slot number (i.e., the total number of elapsed timeslots since the network establishment), $N_{c}$ is the number of available channels, mod is the modulo operation and $F_{\mathrm{mapp}}$ is a lookup table that contains a predefined sequence of channels.

A sensor node is then able to join a TSCH network after successfully receiving an Enhanced Beacon frame containing the following set of information: (i) the timeslot duration; (ii) the number of timeslots in a slotframe; (iii) synchronization-related data; (iv) the channel hopping sequence [6]. Each TSCH timeslot can be of six different types; namely [23]: TxDataRxAck (unicast transmission), TxData (broadcast transmission), RxDataTxAck (unicast reception), RxData (broadcast reception), Idle (indefinitely waiting for a frame) and Sleep (sleep mode).

**Figure 2:**
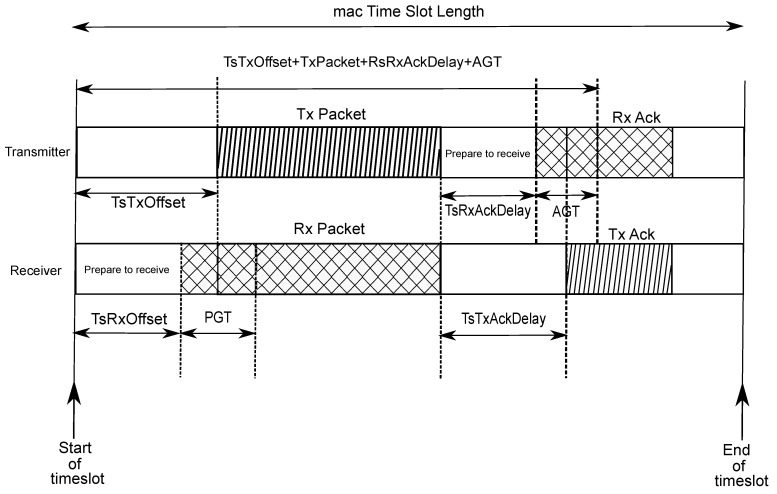
Timeslot template for a TxDataRxAck ↔ RxDataTxAck transaction. (Adapted from [6]).

Figure 2 illustrates the unicast communication process by illustrating the TxDataRxAck ↔ RxDataTxAck transaction.

In the aforementioned transaction, the receiver initially waits the time period TsRxOffset and then turns its radio on. The upcoming phase is the Packet Guard Time (PGT), which lasts for a predefined amount of time, and has the purpose of encompassing possible synchronization issues. Note that the definition of PGT establishes a trade-off between error probability (which is reduced for large values of PGT) and energy consumption (which *increases* for large values of PGT). In case a frame does not arrive within the PGT period, the device goes to the sleep mode for the remainder of the timeslot. However, if a valid packet is received, the node waits TsTxAckDelay after receiving the last byte of the frame, and then becomes active to send an Acknowledgment (ACK) message to the transmitter.

The transmitter node, in turn, initially expends TsTxOffset preparing the data to be transmitted and properly configuring the radio parameters. After that, the radio switches on and starts sending the packet. After the transmission, there is a period of TsRxAckDelay where the transmitter is preparing to receive the ACK from the receiver node. Due to potential synchronization errors, there is also a transmitter-side Acknowledgment Guard Time (AGT), where the transmitter keeps waiting for the incoming ACK.

Usually, due to interference and consumption concerns, sensor nodes in WSNs operate under low duty-cycle policies, typically lower than 1% [23,26]. Thus, one could expect the devices to frequently be either in the Sleep or the Idle modes. As a result, regardless the fact that the power employed in Sleep/Idle modes is considerable lower than that in the active modes, the large amount of time spent in such states leads to an energy consumption that cannot be neglected [23]. Moreover, since the receivers do not know exactly when the transmitter will send a packet, they are expected to keep their radios active during the entire RxDataTxAck and RxData windows upon being assigned to those states, awaiting the predetermined PGT guard time. Thus, it becomes important to model the influence of PGT on both energy consumption and reliability, such that this parameter can be properly adjusted aiming at meeting the application requirements.

## 3. Energy Consumption Model

The availability of an analytical energy consumption model is an important feature that can guide network designers to properly define and adjust the network parameters, subjected to the application requirements. In a practical WSN implementation, however, although the energy consumption is fundamentally dependent on the MCU and the radio consumptions, the overall consumption model varies depending on the platform in use. In this sense, even though recent works have modeled the energy consumption of a TSCH-based network [22,23], their proposed models are valid to the OpenWSN OS only, not accurately estimating the consumption of a Contiki OS TSCH implementation running on a CC2650 hardware platform, as will be seen in the results presented in Section 4.

It becomes then important to evaluate the energy consumption of a Contiki OS-based TSCH network, since it is widely adopted in the WSNs scope [5,24].

### 3.1. Energy Consumption of a CC2650 Running Contiki OS

Contiki OS has a TSCH and 6TiSCH implementation [5], a full low-power IP networking (6LoWPAN, RPL, CoAP and MQTT) and tools for software-based energy estimation, namely Energest and Powertrace [25]. Energest tracks the time the hardware components become active, such as radio or MCU, and Powertrace is responsible for reporting those values. Upon having knowledge about the power consumption of each component, one can then estimate the overall device energy consumption.

**Figure 3:**
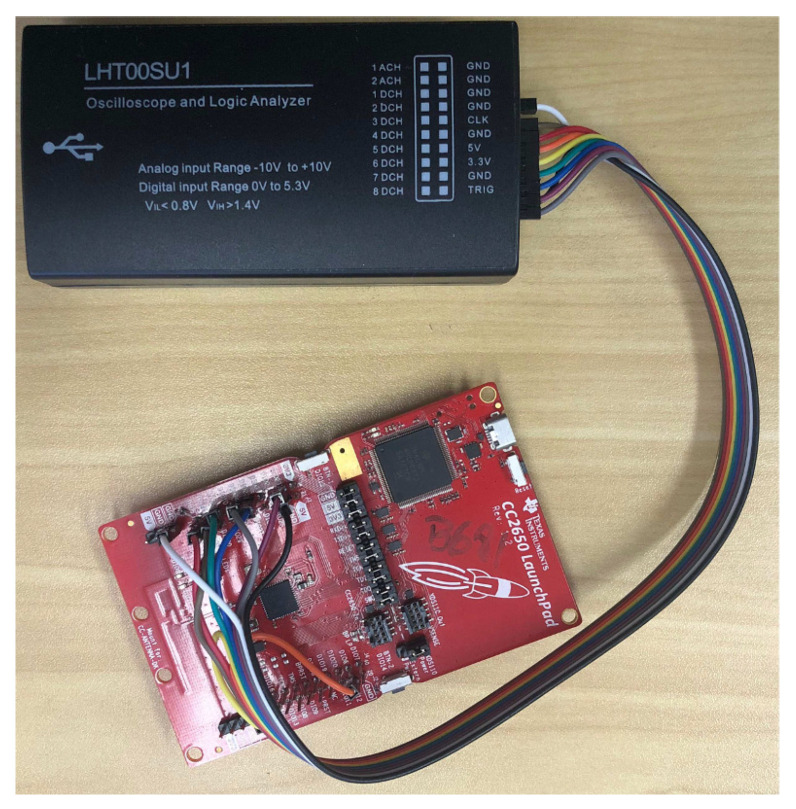
CC2650 Launchpad connected to the Logic Analyzer.

Aiming at obtaining an analytical model for the power consumption, one needs to assess the timing of each hardware component for every type of slot. We then develop a General Purpose Input/Output (GPIO) state logic inside the TSCH DEBUG Macros from the Contiki TSCH implementation, which can be tracked by connecting a logic analyzer to the CC2650 (see Figure 3).

The set S of all the possible logic states is shown in Table 1. Note that each state∈S has an associated number, which is equivalent to the binary number represented by the output pins.

We also define the set of slot types as T={RxDataTxAck,RxData,RxIdle,TxDataRxAck,TxData,Sleep}, such that the average power consumption for a given type∈T is calculated as

(2) $${\bar{P}}_{type}=\sum_{mode\in M}\frac{E_{mode,type}}{T_{\mathrm{slot}}},$$

where M∈{Rx,Tx,CPU,Idle} is the set of consumption modes, $T_{\mathrm{slot}}$ represents the total time of a TSCH time slot and $E_{mode,type}$ corresponds to the energy consumed for a given mode and slot type, being calculated as

(3) $$E_{mode,type}=V\;I_{mode}\sum_{state\in S}T_{type,state,mode},$$

where *V* is the input voltage, $I_{mode}$ is the current drawn at each mode and $T_{type,state,mode}$ is the time spent at each mode for a given state and type pair. Note that a given type does not necessarily have all the states from S neither necessarily operates under all the modes from M. In this situation, $T_{state,mode}$ is set to zero.

Considering that type(t) represents the type whose *t*-th time slot has been assigned to, one can calculate the average power consumption for each mode as

(4) $${\bar{P}}_{mode}=\frac{1}{Q}\sum_{t=0}^{Q-1}\frac{E_{mode,type(t)}}{T_{\mathrm{slot}}},$$

where *Q* is the total number of time slots.

**Figure 4:**
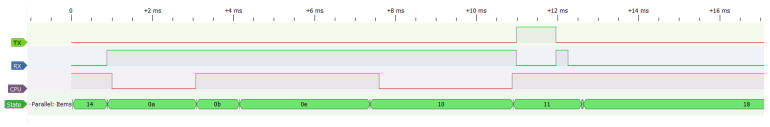
Example of a RxDataTxAck slot captured in the Logic Analyzer Software.

Figure 4 illustrates the procedure adopted to measure the timing properties of a given type, in this particular case a RxDataTxAck, which consists of computing the timing of each mode within the states belonging to that type. Since there is a deviation between different measures of the same 3-tuple (type,state,mode) due to reasons, such as multiple code branches (i.e., different execution paths) and variable duration of an operation (e.g., waking up the radio), we performed several different measures per (type,state,mode) aiming at obtaining an average value.

The average measured values for all the possible combinations of (type,state,mode) are presented in Table 2 and Table 3, always for $T_{\mathrm{slot}}=15$ ms. The sets of states within each type∈T is presented in Table 4, sorted in chronological order of occurrence.

**Figure 5:**
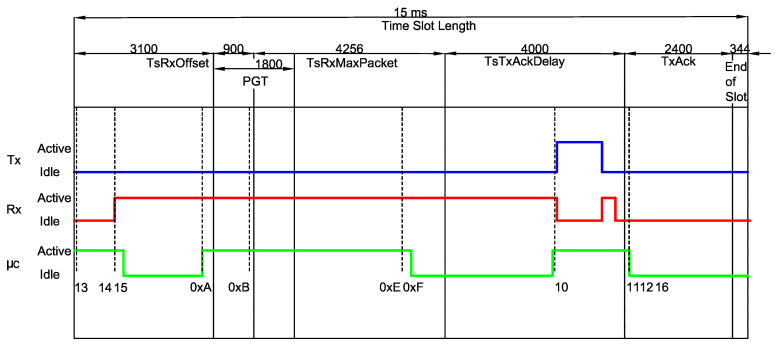
A RxDataTxAck for $T_{\mathrm{slot}}=15$ ms. The timing of each mode within a state can now be computed and a comparison between the standard timeslot model defined by the IEEE 802.15.4e and the measured timing is shown.

It is worth mentioning that such values are significantly different from the values obtained in previous works. Such a difference is depicted in Figure 5, which compares the values measured in this work to the standard timeslot model defined by the IEEE 802.15.4e amendment, as previously shown in Figure 2. For instance, the event 0xA shows that the CPU becomes active before expected (at the end of the TsTxOffset period). Moreover, the receiver also becomes active far before the start of the PGT period, which leads to considerable differences in the overall energy consumption calculations.

As mentioned in Section 1, the nodes need to send (TxData) and receive (RxData) EBs periodically in order to maintain synchronism. Increasing the Guard Time leads to higher energy consumption, since it requires the receiver to be active for a longer period. Usually the receiver and transmitter power consumptions are nearly on the same order [4], being then appropriate to turn off the radio whenever possible. However, the probability of losing an EB increases when the Guard Time is reduced. In practice, depending on the clock drift and the EB interval, there is a lower bound for the Guard Time, such that reducing this bound even further would bring benefits from the energy efficiency’s perspective.

### 3.2. The Guard Beacon Strategy

Aiming at reducing the Guard Time without compromising the synchronization, the so-called Guard Beacon scheme was proposed [9]. This strategy consists of sending a burst of several beacons instead of just a single beacon. The optimal number of beacons is shown to be [9]

(5) $$N_{opt}=\left\lceil \sqrt{\frac{t_{a}P_{l}}{T_{b}P_{s}}}\right\rfloor ,$$

where ⌈·⌋ the nearest integer rounding function, $t_{a}=K\sqrt{T_{s}^{2}\sigma_{f}^{2}+\sigma_{\tau }^{2}+\sigma_{\theta }^{2}}$, $T_{s}$ is the beacon interval, $\sigma_{f}$ is the estimated deviation of clock drift rate, $\sigma_{\tau }$ is the estimated deviation of message delivery delay, $\sigma_{\theta }$ is the estimated deviation of clock offset, $P_{l}$ is the power consumption for idle listening, $T_{b}$ is the beacon duration and $P_{s}$ is the power consumption for data transmission.

The sending time of the *n*th beacon, with $n\in \{1,\ldots ,N_{opt}\}$, is given by [9]

(6) $$x_{n}^{\prime }=\sqrt{2}\;\sigma_{e}{\mathrm{erf}}^{-1}\left(\frac{2n}{N_{opt}}-1\right),$$

where ${\mathrm{erf}}^{-1}\left(\;\cdot \;\right)$ corresponds to the inverse error function.

According to [9], the GB strategy can reduce the synchronization power consumption by ~40%. This motivates us to implement such a strategy over the TSCH beaconing system [27]. Since the beacons required by GB do not carry any additional payload, our implementation considers the use of only two bytes per beacon: one to identify the frame as a beacon and the second one to carry the beacon number.

However, due to setup-related issues, in our implementation the number of adopted beacons is fixed and equal to 3 GBs (Even though the time spent to send one byte is 32 μs, the radio API only returns from a transmission after ~500 μs. Since the standard Guard Time for a 15 ms timeslot is 1800 μs, the number of beacons is then upper limited to 3 GBs). Although this number does not necessarily represent the optimal number of GB from (5), we will show in Section 4 that it provides significant energy savings when compared to the Single Beacon strategy.

**Figure 6:**
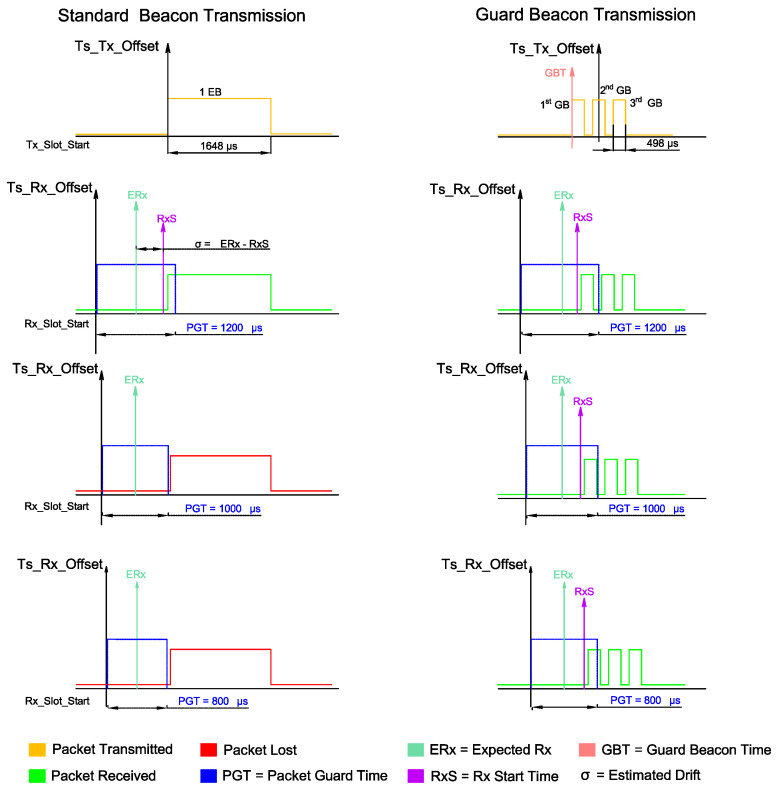
GB Strategy Transmission detailed. The 1st GB is sent prior to TsTxOffset by a Guard Beacon Time (GBT) amount of time. The receiver has an increased probability of receiving one of the GBs even when PGT is small. The estimated drift is calculated as the difference between the Expected Rx Time (ERx) and the Rx Start Time (RxS) so that the receiver can synchronize itself.

Another particularity of our proposal is to anticipate the transmission of the first GB, in order to increase the probability that at least one out of the three GBs is received by all the (multiple) nodes of the network. The idea behind this proposal comes from fact that the clock drifts of a dense network may occur in opposite directions (i.e., +10 ppm for a node and −12 ppm for another node with respect to the network clock source, for instance). Figure 6 illustrates the GB implementation.

Similarly to Section 3.1, we compute the timing for each mode within a state for the types belonging to the GB-based set of types $T_{\mathrm{GB}}=\left\{\mathrm{RxGB},\mathrm{TxGB}\right\}$. The results are also presented in Table 2 and Table 3. Thus, the global average power consumption of a TSCH Network with the Guard Beacon strategy can be calculated with the sum of (4) for every mode∈M and every $type\in T\cup T_{\mathrm{GB}}$. It is worth mentioning that, if a platform different from the CC2650 running the Contiki OS is considered, the only adaptation needed is a new measurement campaign, in order to obtain the values from Table 2 and Table 3 for the new hardware.

**Figure 7:**
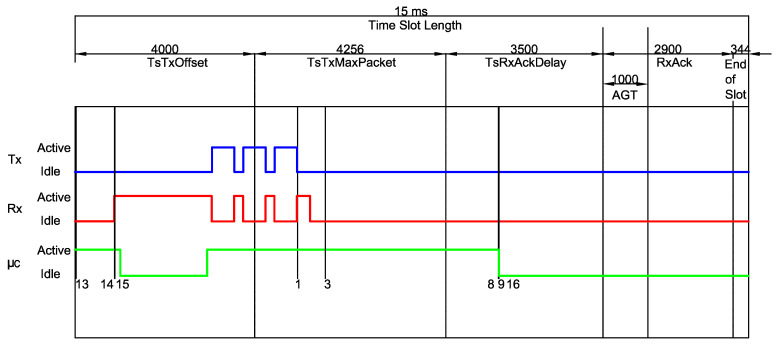
A TxGB slot for $T_{\mathrm{slot}}=15$ ms. As in Figure 5, the timing of each mode within a state is computed and a comparison between the standard IEEE 802.15.4e timeslot model and the measured timing is shown.

**Figure 8:**
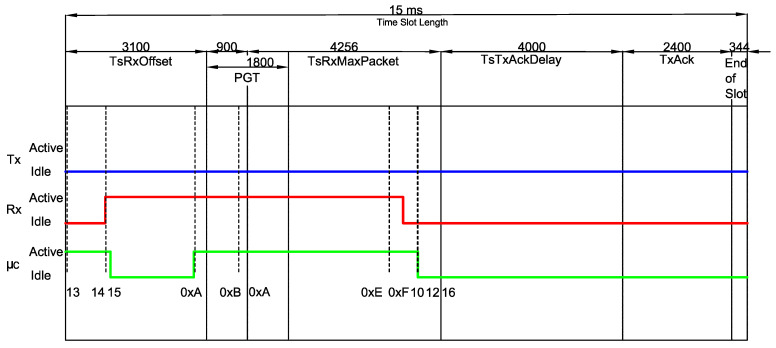
A RxGB for $T_{\mathrm{slot}}=15$ ms. As in Figure 5 and Figure 7, the timing of each mode within a state is computed and a comparison between the standard IEEE 802.15.4e timeslot model and the measured timing is shown.

Additionally, in Figure 7 and Figure 8, a comparison between the standard timeslot model defined by the IEEE 802.15.4e and the measured timing is shown for TxGB and RxGB slots, respectively.

Regarding the scalability of our proposal, the effect of increasing the number of nodes can be neglected for the following reasons: (i) the frequency of Enhanced Beacons is the same for our scheme and the standard TSCH implementation. The difference is that instead of sending one EB we send three GBs on the same timeslot. Thus, our scheme is as scalable as the standard TSCH implementation; (ii) despite an increase in the network traffic, the energy consumption model would not be affected by increasing the number of nodes. Note that the energy consumption model takes into account the number of received and transmitted frames, such that it remains valid regardless the number of nodes.

After closely investigating the outcomes of the entire set of modes and slot types, we realize that an analytical model based solely on the IEEE 802.15.4e standard does not encompass some unexpected behaviors, such as:

1. In a TxData or TxDataRxAck slot, the radio was supposed to enter in a sleep mode after finishing the packet transmission; however, it remains active for approximately an additional period of 370μs;
2. Although there is no incoming Ack in a TxData slot, the receiver remains active for about 298μs;
3. Upon receiving a packet, the receiver unnecessarily remains active for extra ~275μs in a RxData slot, regardless of the size of the received packet;
4. The CPU remains active after the end of the timeslot for approximately 11.7ms in the TxDataRxAck timeslot and about 9.76ms for RxDataTxAck.

Thus, incorporating such odd behaviors into our energy consumption model plays an important role towards improving its accuracy, as we show in Section 4.

## 4. Performance Evaluation

This section presents some analytical and experimental results obtained from a 2-node Contiki TSCH Network using Texas Instruments CC2650 Launchpads. The simulation parameters are given in Table 5.

The two-node network generates a log with information regarding the TSCH operation, which gives us information about the total number of timeslots, and the number of slots of each type, which can be used to calculate the analytical global average power consumption from (4). The log also contains the Contiki’s Powertrace and Energest tools’ data, so that one can calculate the numerical global average power consumption. In what follows, two different scenarios are evaluated: the first one without the GB strategy while varying the EB period (TS); the second one with the Guard Beacon strategy with the same range of EB Periods.

**Figure 9:**
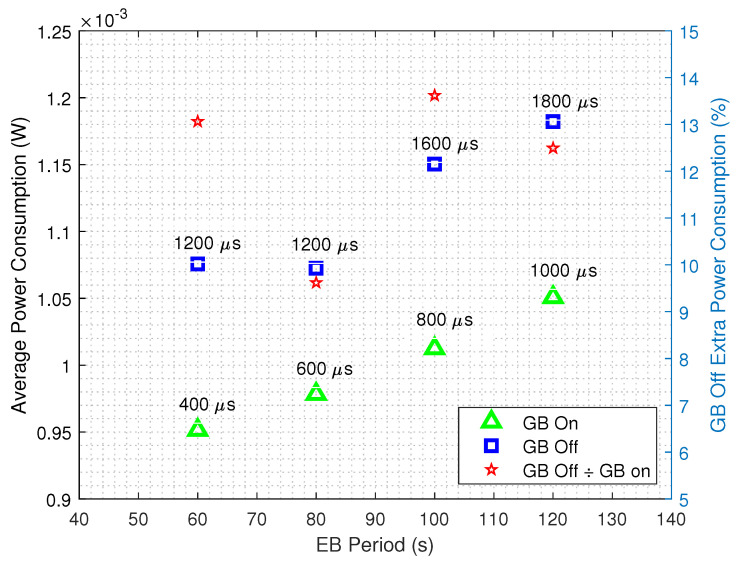
Average power consumption as a function of the EB transmission periods for the GB-disabled and GB-enabled scenarios, while considering the minimum achievable guard times. The right axis shows the amount of extra power spent by the standard TSCH implementation.

The global average power consumptions for the two aforementioned scenarios while considering different EB transmission periods and the minimum achievable guard times are shown in Figure 9. As expected, it can be seen that the GB strategy improves the energy efficiency for all the EB transmission periods, since it can operate at lower guard times when compared to the standard Contiki’s TSCH implementation. On the right axis, the amount of extra power spent by the standard TSCH implementation is shown. For instance, when TS=60 s and the GB strategy is not in use, 13.05% more power is needed.

**Figure 10:**
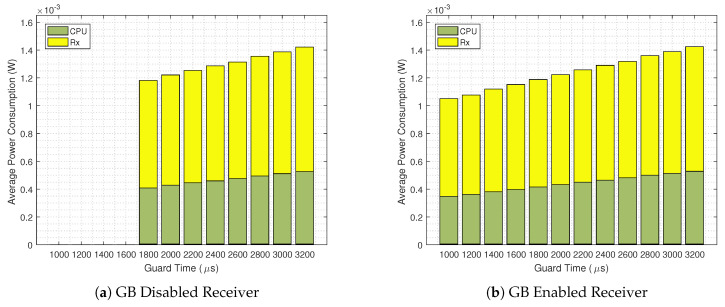
Detailed average power consumption for GB disabled (**a**) and GB enabled scenarios (**b**) for TS=120 s.

Figure 10 shows the detailed average power consumption of mode∈{Rx,CPU} for TS=120 s. The consumptions for mode∈{Tx,Idle} the are barely visible in this scale, so that we opt for omitting their legend. As one could expect from inspecting Figure 9 for TS=120s, the lowest possible power consumption occurs for the GB enabled scenario, with a Guard Time of 1000 μs. For a GB disabled scenario the most energy efficient scenario for the same TS occurs at 1800μs, while consuming 12.67% more power than when the GB technique is in use. It can be seen that for the same guard times, both strategies have roughly the same CPU, Rx and overall power consumption. Again, the key factor that improves the energy efficiency is the lower guard times achieved by the GB enabled scenario.

**Figure 11:**
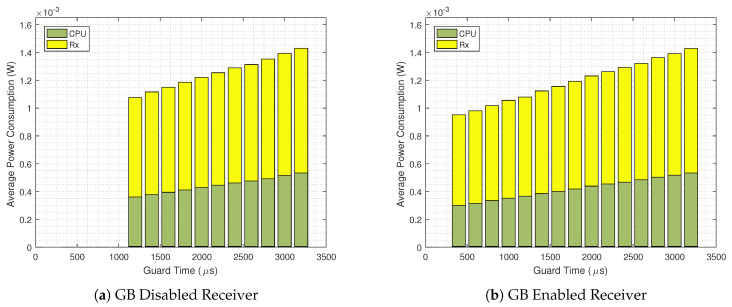
Detailed average power consumption for GB disabled (**a**) and GB enabled scenarios (**b**) for TS=60 s.

The same analysis is shown in Figure 11, for TS=60 s, where the standard TSCH implementation consumes 13.05% more energy than the GB enabled scenario. Moreover, as seen in Figure 9 for TS=60s, with the GB technique the system can operate with a 400 μs guard time, which results in an average power consumption of less than 1 mW, the most energy efficient among all the analyzed scenarios.

**Figure 12:**
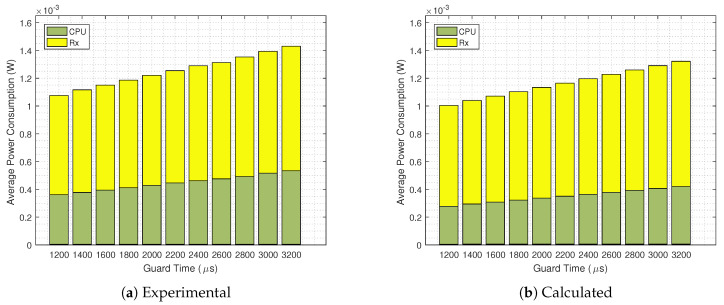
Experimental (**a**) and calculated (**b**) average power consumption for a GB disabled and TS=60 s scenario.

**Figure 13:**
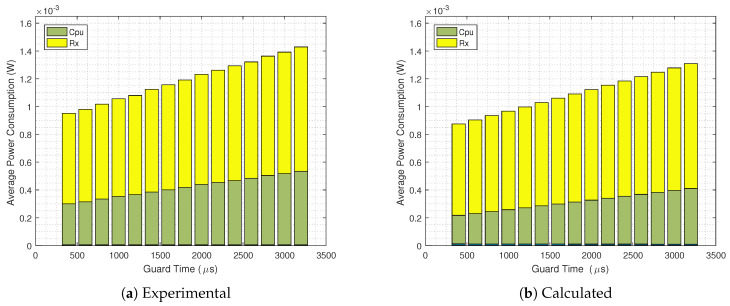
Experimental (**a**) and calculated (**b**) average power consumption for a GB enabled and TS=60 s scenario.

Now, aiming at evaluating the accuracy of our analytical energy consumption model, we compare it with practical results. In Figure 12 we present the detailed average power consumption of mode∈{Rx,CPU} for TS=60 s without the GB strategy implemented. It can be seen that there is a good match; however, the calculated overall power consumption is lower than the results obtained from the Contiki Powertrace tool. The same analysis is done in Figure 13, now with the GB technique in use. Again we see a good match, but with lower power consumption values obtained from the analytical equations.

**Figure 14:**
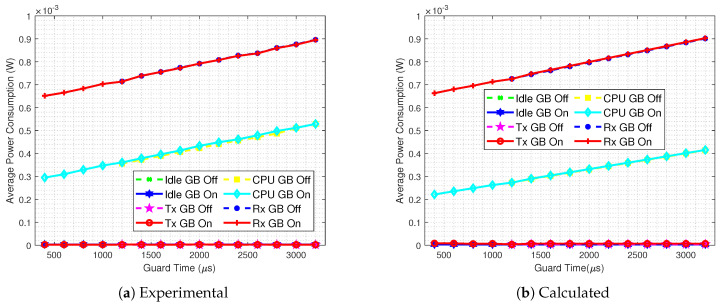
Experimental (**a**) and calculated (**b**) detailed average power consumption comparison between GB enabled and GB disabled scenarios, for TS=60 s

In order to investigate the differences from Figure 12 and Figure 13, the analytical and practical results of every mode for TS=60 s are presented in Figure 14, for both Guard Beacon-enabled and disabled scenarios. We can draw some conclusions:

1. Corroborating the results from Figure 10 and Figure 11, the power consumptions of each mode for GB On and Off scenarios are almost the same;
2. When the Guard Beacon strategy is implemented, energy savings can be achieved by considerably reducing the guard time;
3. The difference between experimental and analytical values is mainly due to the CPU power consumption, since the analytical model does not encompass the CPU states outside the TSCH code.

**Figure 15:**
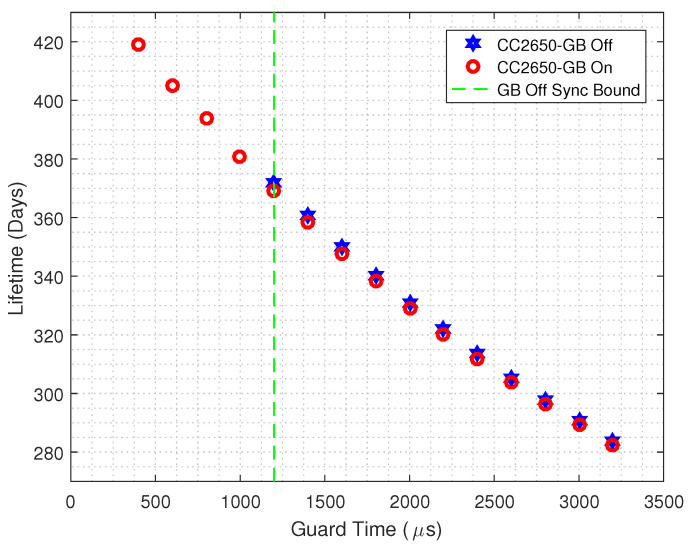
Estimated Lifetime comparison for the CC2650 Launchpad when considering both GB enabled and GB disabled scenarios and a 3000 mAh 3 V battery.

Finally, in order to illustrate the energy savings, in Figure 15 we plot the lifetime of a TSCH-based CC2650 Launchpad platform, powered by a 3000 mAh 3 V battery, for both GB enabled and GB disabled strategies. The same guard time interval is considered. It can be seen that, to the left of the dashed vertical line, only the GB enabled nodes can operate. Thus, the GB strategy can improve the lifetime by 12.93%, which means that a node would operate up to 48 days more when compared to a TSCH node that does not employs the GB scheme.

## 5. Conclusions

In this work we proposed a guard time reduction for the Contiki OS TSCH implementation by employing the Guard Beacon strategy. Additionally, an analytical model for the nodes’ power consumption was derived. We compared the results obtained from the analytical equations and from the Contiki Powertrace tool, which supported the proposed model. Although the equations cannot predict the CPU power consumption outside the TSCH code, it was shown that the model has great accuracy for all the analyzed scenarios. The GB strategy improved the TSCH energy efficiency, since the power consumptions of each mode for GB On and Off scenarios are almost the same, but a guard time reduction up to 66.7% is provided, so that without the strategy the nodes would consume up to 13.05% more power. In terms of battery lifetime, the Guard Beacon approach provided a 48 days extension when considering a 3000 mAh 3 V battery, or 12.93% improvement on the lifetime.

As future work, the impact of the Guard Beacon strategy on the TSCH rendezvous process can be assessed, aiming at reducing the network joining time, which could lead to important energy savings. Additionally, the Guard Beacon strategy could be added to the schemes proposed in [8,14], increasing the energy efficiency even further. Moreover, multi-hop TSCH networks could benefit both from the schemes proposed in [11,13] and the Guard Beacon strategy, so that even lower guard times could be achieved, as well as lower EB sending rates.

## Figures and Tables

**Table 1:** Set S: Debug Logic States.

State	Number	State	Number
TX_INIT	0x01	RX_IDLE_RX_OFF	0x0D
TS_TX_OFFSET_AFTER_TRANSMIT	0x03	PACKET_DETECTED	0x0E
TS_RX_ACK_DELAY	0x04	PACKET_RECEIVED	0x0F
TS_ACK_WAIT	0x05	RX_OFF_AFTER_PACKET_RECEIVED	0x10
ACK_RECEIVED	0x06	RX_ACK_SEND	0x11
RADIO_OFF_AFTER_ACK_RECEIVED	0x07	RX_END	0x12
RADIO_OFF_END_TX_SLOT	0x08	SLOT_START	0x13
TX_END	0x09	SLOT_START_TURN_RADIO_ON	0x14
RX_INIT	0x0A	SLOT_START_RADIO_IS_ON	0x15
TS_RX_OFFSET	0x0B	SLOT_END	0x16
RX_IDLE	0x0C	SLOT_OPERATION_END	0x18

**Table 2:** Average active times in μs for reception slots.

Type		RxDataTxAck			RxData			RxGB			RxIdle		
	Mode	CPU	Tx	Rx	CPU	Tx	Rx	CPU	Tx	Rx	CPU	Tx	Rx
State													
0x0A		139.37	0	2854.37−PGT/2	132.12	0	2854.37−PGT/2	125.5	0	2854.37−PGT/2	129.12	0	2854.37−PGT/2
0x0B		PGT/2+352	0	PGT/2+352	PGT/2+352	0	PGT/2+352	963.62	0	963.62	PGT+515	0	PGT+515
0x0C		-	-	-	-	-	-	-	-	-	495.87	0	166.37
0x0D		-	-	-	-	-	-	-	-	-	4.75	0	0
0x0E		34.86N	0	34.86N	34.86N	0	34.86N	3310.25	0	3310.25	-	-	-
0x0F		4.87	0	4.87	4.87	0	4.87	4.87	0	4.87	-	-	-
0x10		3582.87	250.37	3582.87	632.75	0	294.37	616.37	0	298.5	-	-	-
0x11		1690.00	978.25	376.87	-	-	-	-	-	-	-	-	-
0x12		5.37	0	0	5.37	0	0	5.37	0	0	5.37	0	0
0x13		29.12	0	0	32.25	0	0	29	0	0	29.12	0	0
0x14		851.62	0	7.37	849.37	0	7.25	852.62	0	7.25	846.75	0	7.5
0x15		6.12	0	6.12	6	0	6	6	0	6	6	0	6
0x16		5.5	0	0	6.25	0	0	5.37	0	0	5.37	0	0

**Table 3:** Average active times in μs for transmission slots.

Type		TxDataRxAck			TxData			TxGB		
	Mode	CPU	Tx	Rx	CPU	Tx	Rx	CPU	Tx	Rx
State										
0x01		32N+658.24	32N+434.24	2836.62	32N+658.24	32N+434.24	2836.62	2133.87	1494.62	2571.87
0x03		132.87	0	3270.62	602.12	0	285.12	618.25	0	279.87
0x04		812.5	0	812.5	-	-	-	-	-	-
0x05		617.75	0	617.75	-	-	-	-	-	-
0x06		5.37	0	5.37	-	-	-	-	-	-
0x07		682.25	0	348.37	-	-	-	-	-	-
0x08		3822.37	0	0	3847.75	0	0	3856.87	0	0
0x09		5.62	0	0	5.5	0	0	5.5	0	0
0x13		32	0	0	26	0	0	26	0	0
0x14		874.12	0	7.5	855.62	0	7.25	855.62	0	7.37
0x15		5.87	0	5.87	5.75	0	5.75	5.75	0	5.75
0x16		6.12	0	0	6.25	0	0	6.25	0	0

**Table 4:** Sets of states within each type∈T.

Type	Set of States
RxDataTxAck	0x13, 0x14, 0x15, 0x0A, 0x0B, 0x0E, 0x0F, 0x10, 0x11, 0x12, 0x16
RxData	0x13, 0x14, 0x15, 0x0A, 0x0B, 0x0E, 0x0F, 0x10, 0x12, 0x16
RxIdle	0x13, 0x14, 0x15, 0x0A, 0x0B, 0x0C, 0x0D, 0x12, 0x16
TxDataRxAck	0x13, 0x14, 0x15, 0x01, 0x03, 0x04, 0x05, 0x06, 0x07, 0x08, 0x09, 0x16
TxData	0x13, 0x14, 0x15, 0x01, 0x03, 0x08, 0x09, 0x16
RxGB	0x13, 0x14, 0x15, 0x0A, 0x0B, 0x0E, 0x0F, 0x10, 0x12, 0x16
TxGB	0x13, 0x14, 0x15, 0x01, 0x03, 0x08, 0x09, 0x16

**Table 5:** Simulation Parameters (Radio CC2650 and CPU ARM^®^ Cortex^®^-M3).

Parameter	Value	Parameter	Value
Number of Nodes	2	$V_{\mathrm{supply}}$	3 V
Slotframe length	7	$I_{\mathrm{Tx}}$ [4]	9.1 mA
$T_{\mathrm{slot}}$	15 ms	$I_{\mathrm{Rx}}$ [4]	6.1 mA
$I_{\mathrm{CPU}}$ [4]	2.93 mA	$I_{\mathrm{RF}\;\mathrm{Idle}}$ [4]	1 μA
$I_{\mathrm{CPU}\;\mathrm{Idle}}$ [4]	1 μA		
